# The Blueprint for Rabies Prevention and Control: A Novel Operational Toolkit for Rabies Elimination

**DOI:** 10.1371/journal.pntd.0001388

**Published:** 2012-02-28

**Authors:** Tiziana Lembo, on behalf of the Partners for Rabies Prevention

**Affiliations:** 1 Boyd Orr Centre for Population and Ecosystem Health, University of Glasgow, Glasgow, United Kingdom; 2 Global Alliance for Rabies Control, Manhattan, Kansas, United States of America; Swiss Tropical Institute, Switzerland

## Introduction

Rabies is a prime example of a neglected tropical disease that mostly affects communities suffering from inequitable health care [1]. The false perception that rabies impacts on society are low is due to case under-reporting and limited awareness of the disease burden [2], [3]. Effective tools for elimination of terrestrial rabies are available [4]. While the sustained deployment of these tools has led to some remarkably successful interventions [5], [6], canine rabies continues to claim lives in rabies-endemic countries and areas of re-emergence, where >95% of human deaths occur as a result of bites by rabid domestic dogs [7], [8]. Control programs targeting dogs can effectively reduce the risk of rabies to humans [3], [9]. However, the design and implementation of such programs still pose considerable challenges to local governments, and a lack of easy-to-use guidelines has been identified as an important reason for this.

Global rabies experts from the Partners for Rabies Prevention have therefore gathered to translate evidence-based knowledge on rabies control into user-friendly guidelines. Existing information obtained from different sources, including previously published guidelines by international health and animal welfare organizations and scientific findings, has been packaged into a novel online document, the Blueprint for Rabies Prevention and Control (http://www.rabiesblueprint.com), which we describe herewith.

## The Website

The ultimate goal of the Rabies Blueprint is to provide relevant authorities and personnel in rabies-affected areas with a standard operating procedure (“Blueprint”) to develop their own programs for preventing human rabies through canine rabies elimination and control of wildlife rabies. This document is not meant to replace national legislation or existing guidelines. Rather, it brings all relevant information into one accessible document to make the design of rabies management programs easier globally, both in areas where rabies is endemic or has been re-introduced.

This toolkit has been developed based on the following principles: (1) create a multi-disciplinary working group combining all disciplines/institutions required for formulating comprehensive guidelines for a “One Health” approach to controlling rabies [10]; (2) build upon research-based material to summarize critical information and provide key take-home messages, with links to more detailed information; (3) provide country-specific examples that address common misperceptions and illustrate successful rabies control programs in a range of settings to encourage appropriate investment and efforts elsewhere; (4) follow a question-and-answer approach, reflecting the most frequently asked questions on rabies prevention and control; (5) use language that is understandable by a wide range of users, including professionals and field personnel; (6) use an openly accessible Internet-based format; and (7) develop a low-resolution website to allow users with low Internet speeds or a mobile phone connection to navigate the system.

**Figure 1:**
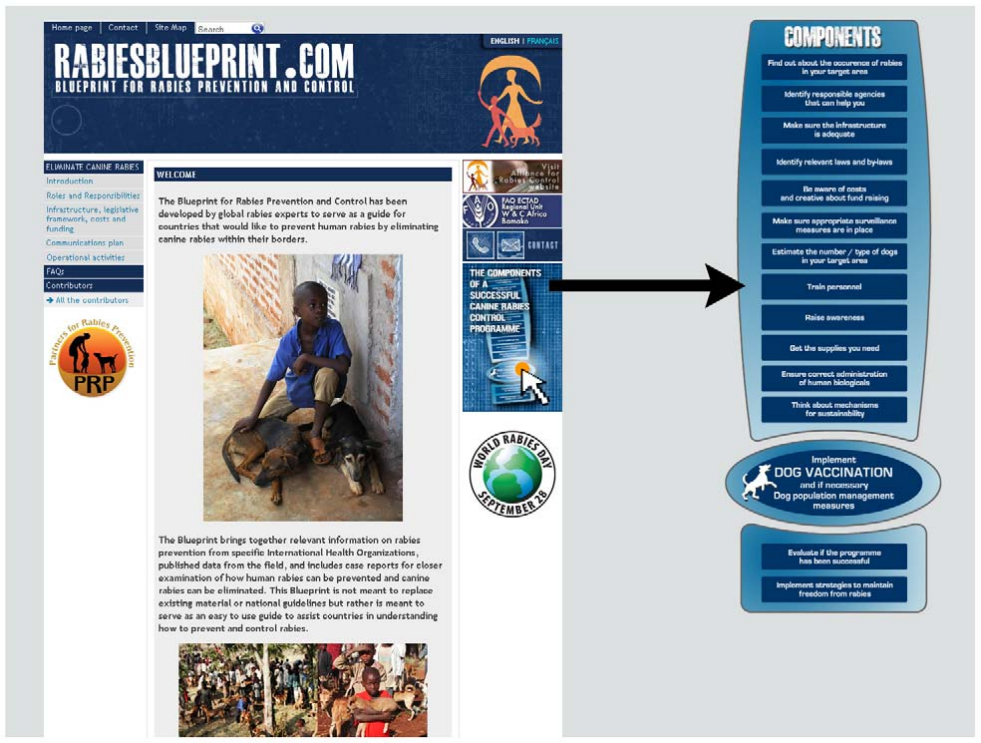
Screenshot of the Rabies Blueprint home page ( http://www.rabiesblueprint.com **).** The sections of the document can be accessed from the left navigation bar, the site map or the diagram to the right, which provides a summary of all components of a successful canine rabies control/elimination program.

The current version of the Rabies Blueprint focuses on dogs as reservoirs, in recognition of the amplified risk to human health from canine rabies. The Rabies Blueprint provides step-by-step guidelines to prevent human rabies by eliminating canine rabies. It addresses key issues that users should consider to achieve this goal, as summarized in the diagram in Figure 1. The document comprises four sections and an introduction, which provides general information about rabies and rabies control strategies. The sections can be accessed from the home page or a simple diagram (http://www.rabiesblueprint.com/spip.php?article119).

The “Roles and Responsibilities” section identifies relevant agencies for all activities related to rabies control programs and defines roles and responsibilities of all sectors involved, emphasizing the importance of building political commitment and establishing cooperation and dialogue amongst all parties. The minimum amount of infrastructure required, legislative frameworks, resource requirements, and funding opportunities are provided in a subsequent section.

Given the importance of enhancing public awareness on rabies prevention and control, the website devotes an entire section (“Communications plan”) to health communication strategies and provides the first rabies communications plan for incorporation into a canine rabies elimination program. This section includes information on how effective communication planning can help raise awareness on rabies; the eight interrelated steps of communication planning; and guidelines for developing country-specific rabies communication plans adaptable to the cultural, political, and behavioral needs of any location.

Finally, the “Operational activities” section contains details of the actual program implementation: epidemiological information required; supplies needed; personnel and training requirements; surveillance mechanisms and diagnostics; operational activities related to the “attack/elimination” component (mass vaccination, dog population management, and human prophylaxis); indicators of success; and requirements to ensure maintenance of a rabies-free status once the disease has been eliminated, including sustainability mechanisms.

Table 1, based on selected popular pages, provides details of the type of information that users can obtain from a number of sections as well as from internal and external links included in each section.

**Table 1:** Examples of the most popular pages of the Rabies Blueprint website based on number of visits recorded since the launch of the website.

Section	Information That Section Provides	Additional Information Provided Through Links	Link to Section
How do I develop a communications plan suitable for my area, region, or country?	Guidelines for development of country-specific communication campaigns for rabies prevention and control adaptable to the local situation	Messages on rabies prevention and control; resource library on health communication planning; interactive planning guide for developing a rabies communication strategy; type, benefits, and limitations of communication channels; and implementation checklist	http://www.rabiesblueprint.com/spip.php?article59
What do we need to do if rabies is re-introduced into an area after a period of absence?	Operational response to contain rabies outbreaks and re-establish freedom from rabies, including agencies to be involved, resources and personnel required, rabies surveillance considerations, and control and prevention measures	Costs associated with rabies control/prevention initiatives and sources of funding; techniques to estimate dog population sizes; protocols for animal catching/handling, vaccination, sample collection and dispatch to the laboratory, and dog population management; laboratory requirements; methods to estimate vaccination coverage; management of detention pounds; and human rabies prophylaxis	http://www.rabiesblueprint.com/spip.php?article106
How does someone start developing a communication plan?	Communication planning framework consisting of eight interrelated steps	Epidemiological information required; KAP studies on rabies and other diseases; message testing materials; and guidelines for developing rabies-specific communication plans	http://www.rabiesblueprint.com/spip.php?article58
What is the Blueprint for Rabies Prevention and Control?	Introduction to the Blueprint and how it works		http://www.rabiesblueprint.com/spip.php?article8
What is rabies and why is it important to control it?	Basic information about rabies	General information about rabies from a range of websites and studies	http://www.rabiesblueprint.com/spip.php?article11
Can rabies be transmitted through food (i.e., by eating milk or meat)?	Basic information on rabies transmission through unconventional routes		http://www.rabiesblueprint.com/spip.php?article73
What techniques are available to estimate the number of dogs?	Guidelines on types of ecological surveys and commonly used census techniques for estimating the number of owned or roaming dogs	Household surveys; human∶dog ratios from a range of settings; methods to assess numbers of roaming dogs (indicator counts, capture-mark-recapture methods, population estimates); and devices to mark dogs	http://www.rabiesblueprint.com/spip.php?article87
Which agencies should be involved in a dog rabies control program?	List of agencies that should be involved in rabies prevention and control initiatives and their roles and responsibilities	Case studies from a range of settings illustrating the role of given agencies in rabies control programs, with an emphasis on intersectoral approaches	http://www.rabiesblueprint.com/spip.php?article23
Why is epidemiological surveillance important and what can we do to enhance it?	What rabies surveillance is, its role in rabies management programs, and commonly used rabies surveillance methods	Evaluation of suspect rabies cases based on clinical signs; simple sample collection methods and supplies for sample collection; diagnostic infrastructure and supplies required; and recommended diagnostic tests	http://www.rabiesblueprint.com/spip.php?article88
Who do we need to train and in what?	Personnel to be involved in rabies management programs and type of training required (e.g., rabies surveillance methods, dog rabies control, human rabies prevention)	Agencies that can provide training; rabies surveillance principles, including rabies diagnostics; recommended human and animal vaccines, and vaccine storage and administration; animal handling, marking devices, vaccination, and euthanasia; legislative frameworks; published guidelines for vaccine production; dog census techniques; and human prophylaxis	http://www.rabiesblueprint.com/spip.php?rubrique20

KAP - Knowledge, Attitude and Practice.

## Impact

Just over one year after the launch of the website in late June 2010, evaluation of its performance indicates that the initiative has had a global impact. Visitors have come from 157 countries/territories and 1,719 cities within the Americas, Europe, Asia, Africa, and Oceania. The number of visits has exceeded 46,000, with consistently >2,000 visitors per month and an average of 80 users daily. Most users (75%) have accessed the English interface. To date, there are several examples of the adoption of this toolkit in a range of countries, such as Uganda, Benin, Afghanistan, Peru, Bolivia, Haiti, and Bali, and evaluation of its impact on rabies control efforts in individual countries is currently being undertaken.

## Conclusions and Future Directions

The multi-disciplinary approach required for the design of effective rabies elimination strategies has linked together veterinarians, public health workers, ecologists, and vaccine producers, which is the first of two important achievements of the Rabies Blueprint initiative. The truly innovative feature of this project is undoubtedly the delivery of a powerful and practical tool directly into the hands of all those with a desire to achieve rabies elimination (Box 1), which makes the Rabies Blueprint a major breakthrough in the global fight against rabies. However, work is still necessary to optimize the website. The expansion from a bilingual (English/French) to a multilingual interface is an essential first step to further increase its global outreach. Translations into Spanish, Portuguese, Russian, Arabic, and Persian have been undertaken, but additional languages will need consideration to reach countries where rabies is an emerging problem (e.g., China and Indonesia). Similarly, although an interactive CD containing a copy of the website has been developed for areas with no or limited Internet access, this is currently only available in English/French. Finally, enduring efforts will be required to ensure incorporation of new/revised recommendations, as well as new tools and ideas, and provide access to additional rabies resources as they become available. An essential next step will be the inclusion of components focusing on wildlife species implicated in rabies maintenance worldwide.

**Box 1. Strengths and Weaknesses**

**Strengths**

- Freely accessible through the Internet (http://www.rabiesblueprint.com)
- All of the most recent information, including case studies, in one single document
- Easy to navigate
- Clear step-by-step recommendations
- Easy to update
- Language/style understandable by a wide range of users, from professionals to field teams
- Applicable from individual exposure to large-scale interventions
- Impact and performance can be monitored
- Bilingual interface
- Upgradable to cover all other reservoir species

**Weaknesses**

- Compared to paper-based guidelines, it requires an Internet connection (website) or access to a computer (CD)
- Potential language barriers
- Potential conflicts with existing national guidelines
- It does not overcome financial and political barriers to canine rabies elimination
